# *In Vitro* Gastrointestinal Digestion of Fresh Cheese: Effect of ω‑3 Fatty Acids and Milk Fat Globule Membrane Enrichment on Nutrient Digestibility and Antioxidant Activity

**DOI:** 10.1021/acs.jafc.5c10823

**Published:** 2025-10-25

**Authors:** Diego Hueso, Samuel Paterson, Victoria Martínez-Sánchez, Blanca Hernández-Ledesma, Antonio Pérez-Gálvez, Javier Fontecha, Pilar Gómez-Cortés

**Affiliations:** † Department of Bioactivity and Food Analysis, 370420 Institute of Food Science Research (CIAL, CSIC-UAM), Nicolás Cabrera 9, Madrid 28049, Spain; ‡ Department of Nutrition and Food Science, Faculty of Pharmacy, Complutense University of Madrid (UCM), Plaza Ramón y Cajal s/n, Madrid 28040, Spain; § Food Phytochemistry Department, Instituto de la Grasa (CSIC), Campus Universitario Building 46, Sevilla 41013, Spain

**Keywords:** cheese, dairy, *in vitro* digestion, ORAC, ABTS, omega-3 PUFA, MFGM

## Abstract

This study aims to provide a first step into understanding the digestion process and potential bioactivity of fresh cheese made from ultrafiltered milk (UFC), focusing on the effects of enrichment with ω-3 polyunsaturated fatty acids (FAs) and a milk fat globule membrane (MFGM) concentrate. To this end, simulated gastrointestinal digestion following the harmonized INFOGEST protocol was carried out on four types of UFC (C: control, W: ω-3 FA enriched, M: MFGM concentrate enriched, and WM: enriched in both ω-3 FA and MFGM concentrate). Neither protein nor lipid digestibility was negatively affected by ω-3 FA or MFGM enrichment, as bioaccessible nutrients were detected after gastrointestinal digestion. Among the cheeses studied, WM exhibited the highest antioxidant capacity, which was associated with the total number of small size peptides and ω-3 FA. Overall, enrichment with ω-3 FA and MFGM concentrate produces cheeses with digestibility similar to that of the control but significantly enhances their antioxidant capacity.

## Graphical abstract

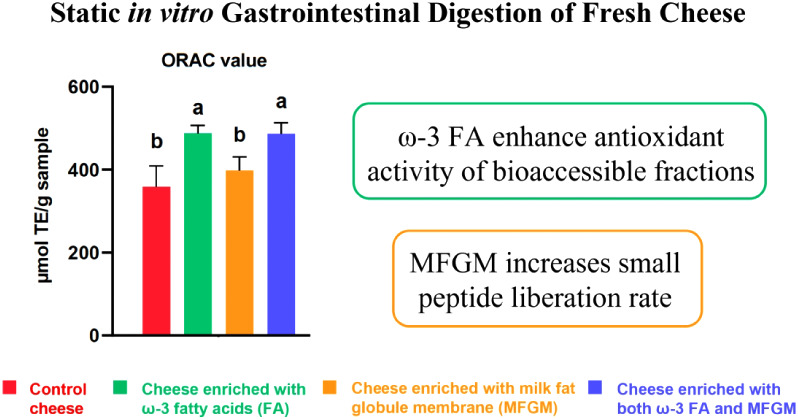

## 1 Introduction

Cheese is a complex protein matrix that provides high levels of nutritionally relevant compounds, including proteins and lipids with both health and technological benefits.^1,2^ In the case of lipids, milk fat primarily consists of globules with a core of triglycerides (TGs) and a trilayer membrane, abundant in polar lipids (PLs) and proteins.^3,4^ Functionality of phospholipids present in the milk fat globule membrane (MFGM), such as phosphatidylcholine (PC), has been described as anticarcinogenic and anti-inflammatory compounds, also preventing coronary heart disease.^5^ However, dietary lipid bioactive compounds are not directly absorbed by the intestinal epithelium and, following lipolysis, require emulsification by bile salts and the formation of mixed micelles.^6^ Likewise, milk proteins must also be hydrolyzed to be absorbed at the small intestine and, digestion-derived peptides that are usually encrypted and inactive within the primary structure, have proven to be beneficial to human health.^7^ Multiple bioactivities have been described for peptides derived from the digestion of caseins and whey proteins, such as antihypertensive, antithrombotic, and antioxidant.^8^ The antioxidant activity is particularly relevant, as it has been correlated with other biological effects and is increasingly used as a preliminary indicator of the bioactive potential of the bioaccessible fraction of functional foods and ingredients.^9–11^

Bioactive compounds can be absorbed after digestion, which can be defined as the physiological process of food decomposition occurring through the gastrointestinal tract to obtain bioaccessible nutrients.^12^ In recent years, bioaccessibility assays have been developed and validated for different types of food matrices to evaluate the release of nutrients and bioactive compounds.^13–15^ The INFOGEST *in vitro* static digestion protocol is the most widely used methodology for bioaccessibility studies and has proven to be effective and suitable for a large number of food matrices.^16^ In addition, it has demonstrated its adaptability to the compositional, processing, and physicochemical characteristics of foods, not only to approximate physiological conditions but also to reduce costs.^17^ This methodology has been used to study the bioaccessibility of nutrients in a wide range of food products, from traditional and unprocessed products such as milk, fruits, or meats to engineered functional foods.^18^

Cheeses have not been an exception in this type of study, with notable examples like the digestion studies by Fang et al., who found that protein and fat were highly bioaccessible at the end of digestion.^19,20^ Asensio-Grau et al. also investigated cheese digestion across various types of commercial cheeses, showing that several factors, such as matrix properties, milk origin, and ripening time, influence lipolysis and protein digestibility.^21^ Regarding fresh cheese digestion, Castaneda and Lee investigated the effect of microstructure and milk homogenization on the bioaccessibility of vitamin D_3_, with its bioaccessibility decreasing when the protein/fat ratio and homogenization pressure increased.^22^ The study by Ribes et al. focused on the influence of food oral processing, bolus characteristics, and digestive conditions on protein digestibility, highlighting the importance of oral processing and the level of fresh cheese matrix breakdown for protein digestibility.^23^ Similar to the present study, the digestion of low-fat Akawi fresh cheese has been previously carried out to determine the biological activity of the bioaccessible fraction.^24^ However, further studies are needed to investigate the impact of other types of fresh cheese matrices on nutrient bioaccessibility and bioactivity, especially when incorporating bioactive compounds.

This study aims to contribute to this field by evaluating the digestion of fresh cheese made from ultrafiltered milk (UFC) and assessing the effects of ω-3 fatty acids (FAs) and MFGM concentrate enrichment, given the well-documented health benefits associated with these bioactive compounds. In addition, the *in vitro* antioxidant activity of the corresponding digests was analyzed as a preliminary, standardized, and comparable assessment of the potential antioxidant capacity of the corresponding digests.

## 2 Materials and Methods

### 2.1 Fresh Cheese Samples

The present study evaluated 4 types of UFC that differed in two main variables: the milk FA profile and the addition of MFGM concentrate during production. Two types of cow’s milk (control and naturally enriched in ω-3 FA through livestock diet) were kindly provided by Feiraco Lácteos S.L. (Ames, Spain) and ultrafiltered in a pilot plant (Perinox, Villarrobledo, Spain) using a 10 kDa OptimaFlow MAX Series membrane (Synder Filtration, Vacaville, CA, USA). MFGM concentrate (Lacprodan, MFGM-10, Arla Foods Ingredients, Viby, Denmark) was incorporated into ultrafiltered milk blends to include 1 g of MFGM per cheese sample (60 g). Samples were denominated as follows: control cheeses without ω-3 FA or MFGM (C), ω-3 FA-enriched cheeses without MFGM (W), MFGM-enriched cheese without ω-3 FA (M), and cheeses enriched in both ω-3 FA and MFGM concentrate (WM). All cheeses were produced in a semi-industrial pilot plant at the Milk Technology Station (Palencia, ES) and characterized through physicochemical analyses (dry matter, total fat, lipid classes, FA profile, total and serum protein, NaCl, and Ca^2+^, Table 1) following the protocols detailed by Hueso et al.^25^

**Table 1:** General Composition (Mean Values ± Standard Deviation) of the Fresh Cheeses Used in the Present Study[Table-fn tbl1fn1]

	C[Table-fn tbl1fn2]	W[Table-fn tbl1fn3]	M[Table-fn tbl1fn4]	WM[Table-fn tbl1fn5]
Dry matter (g/100g)	24.46 ± 0.16^b^	24.61 ± 0.04^b^	25.31 ± 0.11^a^	25.15 ± 0.17^a^
Total fat (g/100g)	5.31 ± 0.05	5.31 ± 0.10	5.16 ± 0.06	5.11 ± 0.30
ω-3 FA (g/100g)	0.45 ± 0.01^b^	0.87 ± 0.08^a^	0.42 ± 0.01^b^	0.85 ± 0.04^a^
Polar lipids (g/100g)	0.077 ± 0.012^b^	0.078 ± 0.034^b^	0.359 ± 0.007^a^	0.389 ± 0.034^a^
Total protein (g/100g)	13.30 ± 0.39^b^	13.07 ± 0.09^b^	14.21 ± 0.15^a^	14.20 ± 0.25^a^
Serum protein (g/100g)	2.94 ± 0.38	3.03 ± 0.35	3.29 ± 0.06	3.21 ± 0.10
NaCl (g/100g)	0.85 ± 0.02	0.84 ± 0.02	0.86 ± 0.03	0.85 ± 0.02
Ca^2+^ (mg/kg)	3984 ± 60^b^	3962 ± 69^b^	4008 ± 87^b^	4190 ± 94^a^

i Means within a row with different superscripts indicate significant differences between samples.

ii C: control cheese.

iii W: ω-3 fatty acid (FA) enriched cheese

iv M: milk fat globule membrane (MFGM) enriched cheese.

v WM: cheese enriched in both ω-3 FA and MFGM.

### 2.2 *In Vitro* Digestion

Simulated *in vitro* gastrointestinal digestion of UFC was performed according to the static digestion protocol INFOGEST 2.0, which follows the common phases of human digestion (oral, gastric, and intestinal phases).^17^ Modifications described by Viera et al. and Kosmerl et al. were also applied to carry out the *in vitro* digestion.^14,26^ The simulated digestion fluids were prepared in advance and frozen (−20 °C) until needed. On the day of the experiment, the solutions were thawed, and both CaCl_2_ and the required enzymes were prepared immediately before use: α-amylase (A3176-porcine pancreas, Sigma-Aldrich), pepsin (P6887-porcine gastric mucosa, Sigma-Aldrich), bile extract (B8631-porcine, Sigma-Aldrich), and pancreatin (P7545-porcine pancreas, Sigma-Aldrich). The *in vitro* digestion process was carried out in triplicate using three replicates of each cheese sample.

Briefly, 5 g of cheese sample were mixed with simulated salivary fluid (SSF), preheated at 37 °C, to obtain a volume of 10 mL at the end of the oral phase. SSF was prepared by mixing an electrolyte stock solution, 25 μL of 0.3 M CaCl_2_ × (H_2_O)_2_, 750 μL of α-amylase solution (1,000 U/mL), and ultrapure H_2_O. The final solution was incubated for 2 min at 37 °C on a rocking platform (VWR rocking platform, 95 rpm). For the gastric phase, 8 mL of warmed simulated gastric fluid (SGF), electrolyte stock solution, 5 μL of 0.3 M CaCl_2_ × (H_2_O)_2_, and the necessary volume of 5 M HCl to adjust the pH to 3.0 were added to the oral bolus, followed by the addition of 667 μL of pepsin (60,000 U/mL) and ultrapure H_2_O to adjust the volume to 20 mL. The gastric phase was run for 2 h at 37 °C and 95 rpm on the rocking platform. For the intestinal phase, 8 mL of warmed simulated intestinal fluid (SIF), electrolyte stock solution, 3 mL of bile salt solution (10 mM), and the necessary volume of 5 M NaOH to adjust the pH to 7.0 were added to each sample, and incubation was continued for 30 min under the same conditions to ensure bile salt solubilization. Afterward, 40 μL of 0.3 M CaCl_2_ × (H_2_O)_2_, 5 mL of pancreatin suspension, and ultrapure H_2_O were added to a final volume of 40 mL. Pancreatin was incorporated into the SIF solution to yield a final trypsin activity of 100 U/mL. The incubation conditions for the intestinal phase (2 h) remained constant from the previous ones. After completion of the gastric and intestinal phases of *in vitro* digestion, 10 mL aliquots were transferred to centrifuge tubes and immediately placed in ice for enzymatic deactivation, as suggested by the INFOGEST 2.0 protocol.^17^ Immediately afterward, samples were stored at −20 °C until recuperation of the micellar phase, which was carried out according to the protocol described by Corzo-Martínez et al. (4000 rpm for 40 min at 20 °C).^27^ After supernatant (SN) and pellet separation, samples were stored at −20 °C until subsequent analyses.

The methodological limitations of this study stem primarily from strict adherence to the INFOGEST protocol. The omission of gastric lipase, justified by its reported lack of necessity in low-fat protein matrices, may have limited the ability to reliably determine lipid classes in the gastric digesta. Therefore, the lipid analysis described below focused solely on the final gastrointestinal digests to assess *in vitro* lipid digestion.

### 2.3 Lipid Analysis

#### 2.3.1 Lipid Extraction

Fat extraction was carried out according to Löfgren et al., with slight modifications.^28^ Briefly, 200 μL of sample was mixed with 1.6 mL of methanol and 2.49 mL of dichloromethane and then stirred with a rotational agitator (OVAN N12E, Badalona, Spain) for 20 min. Then, 1 mL of 20 mM acetic acid was added; the mixture was stirred and centrifuged at 3200 rpm for 5 min at 4 °C, and the bottom organic phase was carefully removed. The upper methanol phase was washed again with dichloromethane, removed, and combined with the prior one. Finally, the total organic phase was passed through a syringe containing anhydrous sodium sulfate and a PVDF filter with a membrane pore size of 0.45 μm (Sigma-Aldrich, Saint Louis, USA). Lipid extracts were collected in amber vials, flushed with nitrogen, weighed, and stored at −20 °C until analysis.

#### 2.3.2 Lipid Classes by HPLC-ELSD

Separation of lipid classes was accomplished in a HPLC system (model 1260; Agilent Technologies Inc., Palo Alto, CA, USA) coupled with an ELSD (SEDEX 85 model, Sedere SAS, Alfortville, France) using prefiltered compressed air as the nebulizing gas at a pressure of 350 kPa at 60 °C; the gain was set at 3. Two columns in series (250 × 4.5 mm Zorbax Rx-SIL column with a 5 μm particle diameter; Agilent Technologies Inc.) and a precolumn with the same packing were used. Before analysis, samples were dissolved in CH_2_Cl_2_ (at 5 mg/mL), and 50 μL were injected after column equilibration at 40 °C. The solvent gradient was as detailed by Castro-Gómez et al.^29^ Samples and standards were analyzed under the same chromatographic conditions, and all the analyses were carried out in duplicate.

### 2.4 Protein Analysis

#### 2.4.1 Electrophoresis

SDS-PAGE was carried out on ultrafiltered milks, UFC, and the corresponding gastric and gastrointestinal cheese digests (SN and pellets) after the centrifugation step. Electrophoresis was performed in the Criterion Cell electrophoresis tank (Bio-Rad, Hercules, CA, USA) using 1× XTMES buffer, prepared by mixing 50 mL of 20× XTMES with 950 mL of Milli-Q H_2_O. The analysis was conducted under reducing conditions using β-mercaptoethanol as the reducing agent, following the method previously described by Paterson et al.^30^ Each sample was diluted in a sample buffer to reach a final protein concentration of 0.8 mg/mL. The molecular weight marker used was Precision Plus Protein Dual Xtra Prestained Protein Standards (Bio-Rad). The gels were stained with Bio-Safe Coomassie G-250 and subsequently washed to obtain and analyze images with the VersaDoc Imaging System gel reader and Image Lab version 6.1 software (Bio-Rad).

#### 2.4.2 Molecular Weight Distribution of Peptides by MALDI-TOF

Preliminarily, the elimination of salts and peptide concentration from the gastric and gastrointestinal digest SNs were carried out using the ZipTip_C18_ ZTC18S096 pipette tips (Merck Millipore, Burlington, MA, USA) following the manufacturer’s instructions. Then, samples were diluted 100-fold in a solution of 50% acetonitrile and 0.1% trifluoroacetic acid, and the analyses were carried out on a MALDI target plate with α-Cyano-4-hydroxycinnamic acid (HCCA) as previously described by Santos-Hernández et al.^31^ Ions were detected in a positive linear mode using the Autoflex Speed and were collected from the sum of 1,000 on average laser shots. A peptide and a protein calibration standard (Protein Calibration Standard I, Bruker Daltonics) were employed for the external calibration of spectra. The monoisotopic peak lists were generated by the FlexAnalysis 3.3 software and were represented in a molecular weight distribution range after the elimination of the peptide’s correspondent to the HCCA matrix and the digestion blank used.

### 2.5 Antioxidant Activity Assessment

#### 2.5.1 ABTS Assay

The ABTS (2,2′-azino-bis­(3-ethylbenzothiazoline-6-sulfonic acid)) radical scavenging activity was determined for each gastric and gastrointestinal digest SN, following the method described by Paterson et al., adapted from the original protocol by Re et al.^30,32^ Briefly, sample serial dilutions (0.25, 0.5, 0.75, 1.0, 1.5, and 2.0 mg/mL) and a Trolox standard curve (25–200 μM) were prepared. Then, 20 μL of PBS (blank), Trolox, and sample dilutions were placed in a 96-well transparent microplate. 180 μL of diluted ABTS radical were added to each well, mixed, and incubated for 7 min at room temperature, measuring absorbance at 734 nm in the Biotek Synergy HT plate reader (Winooski, USA). Trolox equivalent antioxidant capacity (TEAC) values were determined in sextuplicate and expressed as μmol of Trolox equivalents (TEs)/g of sample.

#### 2.5.2 ORAC Assay

The oxygen radical absorbance capacity (ORAC) assay was performed on each gastric and gastrointestinal digest SN following Hernández-Ledesma et al.^33^ Briefly, the reaction was carried out in a black 96-well microplate at 37 °C in 75 mM phosphate buffer (pH 7.4). The final assay mixture in each well (200 μL) contained fluorescein (117 nM), 2,2’-Azobis­(2-amidinopropane) dihydrochloride (AAPH, 14 mM), and either antioxidant (Trolox, 0.2–1.6 nmol) for the standard curve or sample diluted in phosphate buffer (0.0312–1 mg/mL). The fluorescence was recorded every 2 min for 120 min at 485 and 520 nm wavelengths of excitation and emission, respectively, with the Fluostar Optima BMG Labtech plate reader controlled by FLUOstar Control version 1.32 R2 software (Ortenberg, Germany). ORAC values were determined in quadruplicate and expressed as μmol TE/g of sample.

### 2.6 Statistical Analyses

The results obtained were analyzed by ANOVA, followed by Fisher’s least significant difference test using the statistical analysis program XLSTAT Premium, version 2018.5.53172 (Addinsoft, Paris, France). Differences were considered statistically significant at *p* < 0.05.

## 3 Results and Discussion

General composition of the UFC before *in vitro* digestion is shown in Table 1. Total fat content was similar in all cheese samples, while ω-3 FA levels were doubled in W and WM cheeses, when compared to cheeses elaborated with control milk (C and M). PL content increased by fourfold in M and WM cheeses due to the inclusion of MFGM concentrate. M and WM also showed a slight increase in total protein content. However, no significant differences were observed in serum protein levels, indicating that the incorporation of MFGM did not add extra whey protein to the UFC.

### 3.1 Lipid Digestion of UFC

All cheeses, both control and experimental, had a fat content ranging from 5.1% to 5.3% (Table 1). In accordance with the INFOGEST 2.0 protocol and the 2022 study by Viera et al., which investigated lipid digestion in various low- and high-fat matrices, gastric lipase was omitted during the gastric phase.^14,17^ This decision was based on the fact that the UFC under study was low-fat, high-protein matrices, for which the addition of gastric lipase is considered unnecessary. However, to better mimic *in vivo* conditions and overcome the limitations of the present study, simulated gastrointestinal lipid digestion models should also evaluate the incorporation of gastric lipase in future research.

Lipid extraction was carried out in the undigested UFC and the supernatants of the final gastrointestinal digests to obtain information about the differential distribution of lipid moieties. Table 2 displays lipid classes of these undigested and digested samples. In the undigested UFC, TG was the predominant lipid class, ranging from 88 to 93 g/100 g of total lipid content. A significant difference was observed among the undigested samples in the polar lipid content. Those cheeses enriched with MFGM (M, 0.36 ± 0.01 g/100 g; WM, 0.39 ± 0.03 g/100 g) contained over 4.5 times more polar lipids than the nonenriched counterparts (C, 0.08 ± 0.01 g/100 g; W, 0.08 ± 0.01 g/100 g, Table 2).

**Table 2:** Lipid Classes (g/100g) of Undigested Fresh Cheeses (C: Control; W: ω-3 Fatty Acid, FA; M: Milk Fat Globule Membrane, MFGM; WM: ω-3 FA and MFGM) and the Supernatants from Their Corresponding Gastrointestinal Digests[Table-fn tbl2fn1]

Undigested sample					Gastrointestinal digest			
Lipid class	C	W	M	WM	C	W	M	WM
TG[Table-fn tbl2fn2]	92.52 ± 0.88^a^	91.01 ± 0.50^b^	88.46 ± 1.09^c^	93.04 ± 0.43^a^	0.08 ± 0.02^d^	0.06 ± 0.06^d^	0.04 ± 0.01^d^	0.06 ± 0.01^d^
DG[Table-fn tbl2fn3]	5.62 ± 0.99^b^	8.51 ± 0.51^a^	8.43 ± 1.50^a^	6.17 ± 0.40^b^	-	0.43 ± 0.11^c^	-	0.59 ± 0.18^c^
MG[Table-fn tbl2fn4]	0.02 ± 0.00^c^	0.02 ± 0.00^c^	0.02 ± 0.00^c^	0.02 ± 0.00^c^	22.47 ± 7.17^ab^	17.50 ± 6.12^ab^	17.96 ± 3.08^b^	27.21 ± 5.36^a^
FFA[Table-fn tbl2fn5]	0.37 ± 0.05 ^c^	0.38 ± 0.05^c^	0.52 ± 0.02^c^	0.39 ± 0.05^c^	77.45 ± 7.17^ab^	82.02 ± 6.24^a^	81.97 ± 3.09^a^	68.97 ± 5.55^b^
GluCer[Table-fn tbl2fn6]	-	-	0.01 ± 0.00	0.02 ± 0.01	-	-	-	-
PL[Table-fn tbl2fn7]	0.08 ± 0.01^b^	0.08 ± 0.03^b^	0.36 ± 0.01^a^	0.39 ± 0.03^a^	-	-	-	-
PE[Table-fn tbl2fn8]	0.04 ± 0.01^b^	0.05 ± 0.00^b^	0.17 ± 0.01^a^	0.17 ± 0.02^a^	-	-	-	-
PI^i^	-	-	0.01 ± 0.01	0.01 ± 0.00	-	-	-	-
PS[Table-fn tbl2fn10]	-	-	0.01 ± 0.01	0.01 ± 0.00	-	-	-	-
PC[Table-fn tbl2fn11]	0.03 ± 0.00^b^	0.03 ± 0.00^b^	0.12 ± 0.00^a^	0.13 ± 0.02^a^	-	-	-	-
SM[Table-fn tbl2fn12]	-	-	0.04 ± 0.00	0.05 ± 0.01	-	-	-	-

i Means within a row with different superscripts indicate significant differences between samples.

ii TG: triglyceride.

iii DG: diglyceride.

iv MG: monoglyceride.

v FFA: free fatty acid.

vi GluCer: glucosyl ceramide.

vii PL: polar lipid.

viii PE: phosphatidyl ethanolamine.

ix PI: phosphatidyl inositol.

x PS: phosphatidyl serine.

xi PC: phosphatidyl choline.

xii SM: sphingomyelin.

After *in vitro* digestion, TGs were almost completely hydrolyzed with lipolysis rates above 99.5% for all cheese samples (Table 2). As a result, FFAs were the predominant lipid class, consistent with previous *in vitro* digestion studies reporting lipolysis rates of 90–100% in solid, protein-rich food matrices.^34^ Although no significant differences were found in FFA from WM gastrointestinal digests (68.97 ± 5.55 g/100 g) compared to the control (77.45 ± 7.17 g/100 g), significant differences were observed compared to those of W (82.02 ± 6.24 g/100 g) and M (81.97 ± 3.09 g/100 g) gastrointestinal digests. This effect might be attributed to the higher calcium concentration observed in WM cheeses (Table 1), as ionic calcium can form insoluble soaps with long-chain saturated FAs.^35^ In this line, it has been previously reported that the bioaccessibility of FFA in Cheddar cheese was affected by calcium levels due to the formation of insoluble calcium soaps.^36,37^ In the case of glycosylated ceramides (GluCer) and PLs, no residual lipids were detected after *in vitro* digestion, indicating complete hydrolysis within the detection limits of the technique. These findings are in alignment with a previous study by Shen et al., in which PL from human milk exhibited the highest hydrolysis rate after 2 h of simulated gastrointestinal digestion.^38^ PLs derived from bovine MFGM share a more similar composition to human PL than other sources, which may confer higher *in vitro* bioaccessibility of PL in MFGM-enriched cheeses.^39^

The bioaccessibility of FA and other lipid moieties in dairy products does not solely depend on their total content, but is also influenced by food structure.^40^ Dairy products exhibit a wide range of structural characteristics due to variations in milk origin (i.e., ruminant species and composition) and processing methods.^41^ In this context, a previous *in vitro* digestion study on dairy products reported that the physical state of the product, whether liquid, such as milk or yogurt, or solid, such as cheese, had minimal influence on the degree of lipolysis.^42^ However, a prior study reported that the native MFGM surrounding milk fat globules inhibited lipolysis and resulted in a slower rate of hydrolysis compared to modified globules coated with caseins instead of MFGM.^43^ This effect was not observed in the present study, even in the MFGM-enriched cheeses, which suggests that the inhibitory effect would be associated with the native structure surrounding fat globules rather than its mere presence in the digestion medium.

### 3.2 Protein Digestion of UFC

#### 3.2.1 Protein Gastric Digestion

The protein profiles of the original concentrated milks, undigested cheeses, and corresponding cheese digests were analyzed through SDS-PAGE (Figure 1A,B), and the percentages of band density are shown in Tables S1 and S2. First, several bands with molecular weights in the range of 14–155 kDa were present in both concentrated milks and undigested cheeses, corresponding to the main proteins that have been previously described in milk: xanthine oxidase/dehydrogenase, lactoferrin, bovine serum albumin, butyrophilin, adipophilin, lactadherins, α_s2_-casein, α_s1_-casein, β-casein, κ-casein, β-lactoglobulin A, β-lactoglobulin B, and α-lactalbumin.^44,45^ Undigested cheeses also presented a characteristic band with a molecular weight around 13–14 kDa that would correspond to para-κ-casein, generated during cheese production.^46^ Most of the bands detected in the undigested cheeses disappeared, and only bands with low molecular weights (13–20 kDa) were present in gastric SNs. These persistent bands corresponded to β-lactoglobulin A, β-lactoglobulin B, and α-lactalbumin, which have been shown to be resistant to pepsin proteolysis during cheese *in vitro* gastric digestion.^47^ Regarding cheese gastric pellets, several bands with molecular weights from 11.2–65 kDa appeared (Figure 1A,B). These bands could correspond to the enzymes α-amylase (62 kDa) and pepsin (38.3 kDa) used in the digestion process, as well as to the partially hydrolyzed casein-derived proteins and fragments. Caseins are naturally hydrophobic proteins that are water-soluble due to only the amphiphilic hydrocolloidal properties of κ-casein. However, after gastric digestion by pepsin, fragments might migrate to the pellets during the centrifugation step.^48,49^

**Figure 1:**
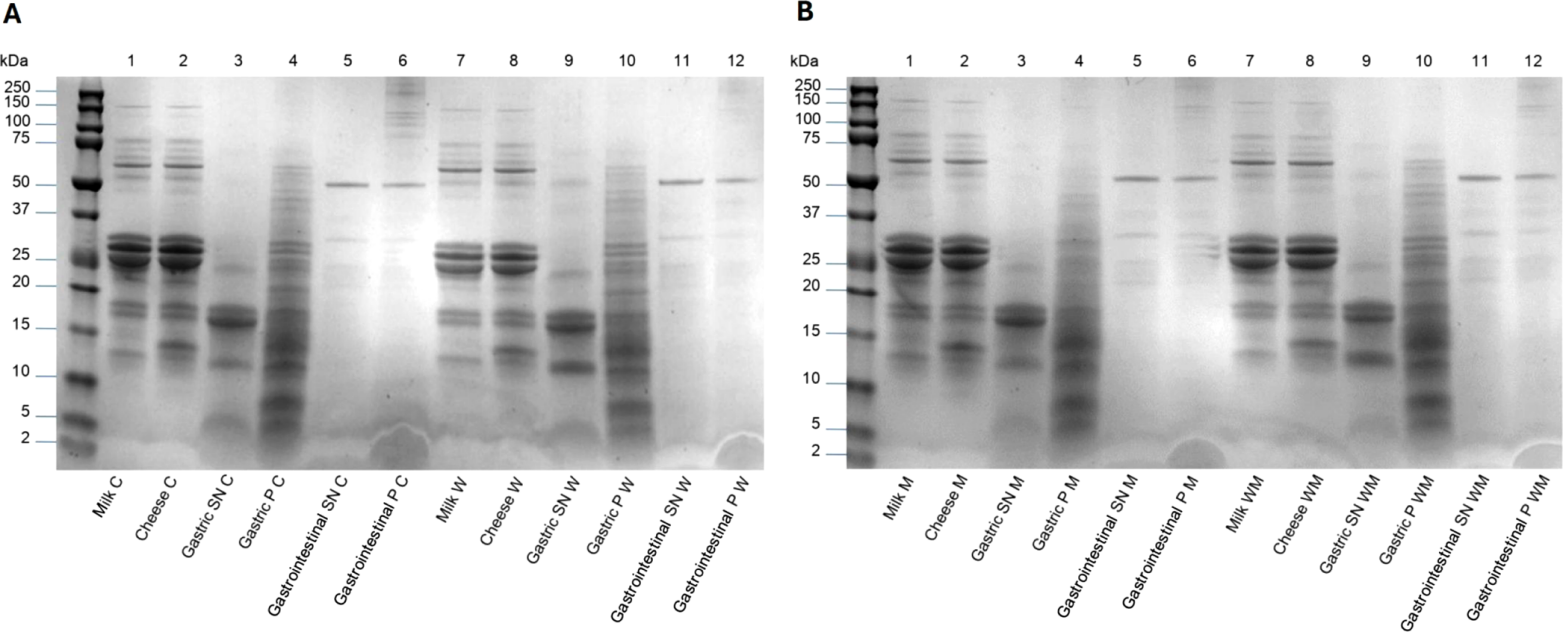
SDS-PAGE analysis of milk, fresh cheese made from ultrafiltered milk, and *in vitro* digests. A) C (control) and W (ω-3 fatty acid, FA) samples. B) M (milk fat globule membrane, MFGM) and WM (ω-3 FA and MFGM) samples. SN: supernatant. P: pellet.

Figure 2 shows the distribution of peptides across molecular weight ranges for peptides released in gastric and gastrointestinal SNs. In general terms, all gastric samples had predominantly peptides between 4900 and 9900 Da and 9900–20000 Da, as reflected in peptide count (Figure 2A) and peptide relative abundance (Figure 2B). Specifically, gastric digests of cheeses enriched with ω-3 FA (W and WM) showed the highest total peptide count (54 peptides), followed by M (51 peptides) and C (34 peptides). Digests from MFGM cheeses (M and WM) showed more peptide relative abundance in the 9900–20000 Da range (82.6%), which contrasted with the rest of the samples (W, 57.2% and C, 38.8%). Additionally, W and WM gastric digests presented unique peptides identified with *m*/*z* values of 507, 533, and 711 (Table 3). Pepsin cleaves peptide bonds in the amino-terminal side of the cyclic amino acid residues (tyrosine, phenylalanine, and tryptophan), breaking the polypeptide chains into smaller peptides.^50^ Given that cheese-derived proteins are known to be susceptible to pepsin activity in the stomach, our findings suggest that, during the *in vitro* gastric phase, the endopeptidase activity of pepsin may have cleaved peptide bonds within the casein matrix.^51^ This enzymatic action likely reduced the cohesive forces maintaining the integrity of the cheese matrix, resulting in partial hydrolysis and facilitating disintegration, which is key for the subsequent intestinal phase.

**Figure 2:**
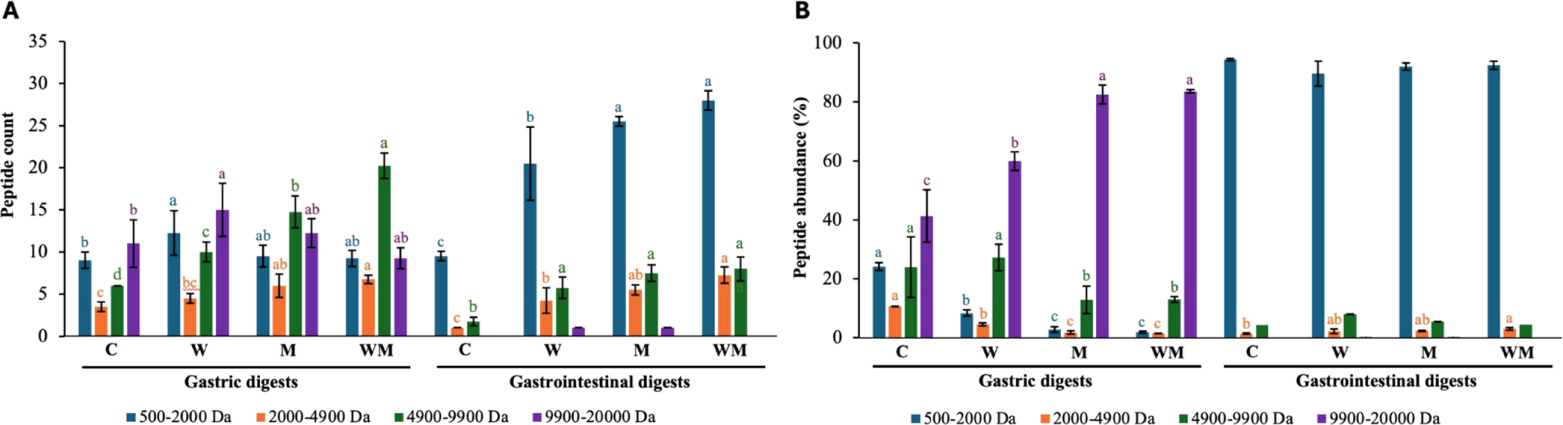
General peptide characterization of supernatants from *in vitro* digests of fresh cheeses made from ultrafiltered milk. A) Peptide count. B) Peptide relative abundance. C: control. W: ω-3 fatty acids (FAs). M: milk fat globule membrane (MFGM). WM: ω-3 FA and MFGM. Superscripts a, b, c, and d indicate significant differences within each digest, gastric and gastrointestinal, according to the size range (Da).

**Table 3:** Peptide Identification (*m/z*) in Supernatants of *In Vitro* Gastric and Gastrointestinal Digests of Fresh Cheeses (C: Control; W: ω-3 Fatty Acid, FA; M: Milk Fat Globule Membrane, MFGM; WM: ω-3 FA and MFGM)

	Gastric digest				Gastrointestinal digest			
*m*/*z*	C	W	M	WM	C	W	M	WM
**503**	-	-	-	-	-	-	-	**+**
504	-	+	+	-	-	-	-	-
505	-	-	-	-	+	+	+	+
**507**	-	**+**	-	-	-	-	-	-
509	-	-	-	-	-	+	+	+
512	+	+	+	+	-	-	-	+
517	-	-	-	-	-	+	+	+
519	-	-	-	-	+	+	+	+
521	-	-	-	-	+	+	+	+
523	-	-	-	-	+	+	+	+
525	-	-	-	-	+	+	+	-
**533**	-	**+**	-	-	-	-	-	-
541	-	-	-	-	-	+	+	-
543	+	-	-	-	+	+	+	+
549	-	-	-	-	+	+	+	+
559	+	-	-	-	+	+	+	+
567	-	-	-	-	+	+	+	+
573	+	+	+	+	-	-	-	-
575	-	+	-	-	-	-	+	+
**583**	-	-	-	-	**+**	-	-	-
604	-	-	-	-	-	+	+	+
606	-	-	-	-	+	+	+	+
615	-	+	+	+	-	-	-	-
637	+	+	+	+	-	-	-	-
641	-	+	+	+	-	-	-	-
**649**	-	-	-	-	**+**	-	-	-
656	-	-	-	-	-	+	+	+
**665**	-	-	-	-	**+**	-	-	-
686	-	-	-	-	-	+	+	+
691	-	-	-	-	+	-	+	+
704	-	-	-	-	+	+	+	+
709	-	-	-	-	+	+	+	+
**711**	-	-	-	**+**	-	-	-	-
**720**	-	-	-	-	**+**	-	-	-
**724**	-	-	-	-	-	**+**	-	-
827	-	-	-	-	-	-	+	+
875	-	+	+	+	-	-	-	-
913	+	+	-	-	-	-	-	-
**915**	-	-	-	-	**+**	-	-	-
927	-	-	-	-	-	-	+	+
**928**	-	-	-	-	-	-	**+**	-
**931**	-	-	-	-	**+**	-	-	-
Peptide count	6	11	7	7	19	19	23	22
Unique peptides^a^	**0**	**2**	**0**	**1**	**6**	**1**	**1**	**1**

a Unique peptides are highlighted in bold.

#### 3.2.2 Protein Intestinal Digestion

Most of the bands visualized in the gastric digest disappeared after the intestinal phase (Figure 1, Tables S1 and S2, Supporting Information), and only bands with molecular weights ranging from 24–55 kDa were identified. These band would correspond to the intestinal enzymes used, such as trypsin (24 kDa), chymotrypsin (27 kDa), pancreatic lipase (52 kDa), and pancreatic α-amylase (55.4 kDa), which have been previously described by SDS-PAGE.^52^ These findings suggest that partial casein gastric hydrolysis was completed during the *in vitro* intestinal phase. In contrast with cheese gastric digests, most of the peptides observed in the gastrointestinal digest SNs were in the range of 500–2000 Da in all samples (Figure 2). The gastrointestinal digest from WM showed the highest number of total peptides (46), followed by those from M (44 peptides), W (36 peptides), and C (22 peptides). Following the same tendency, WM and M presented more peptides in the 500–2000 Da range (28 peptides; see Figure 2A). More specifically, peptides with *m*/*z* values of 504, 512, 573, 615, 637, 641, 875, and 913 detected in the gastric SNs decreased or disappeared in the intestinal digests. Intestinal enzymes also generated new and smaller dipeptides and tripeptides (e.g., 505, 509, 517, 519, 521, 523, 525) that appeared only in the gastrointestinal digests (Table 3).

Trypsin specifically cleaves peptide bonds on the carboxyl side of lysine and arginine residues, whereas chymotrypsin cleaves peptide bonds on the carboxylic side of aromatic or hydrophobic amino acid residues. As a result, peptides released by chymotrypsin often have histidine, alanine, valine, leucine, tyrosine, phenylalanine, tryptophan, or proline at the C-terminal position. Therefore, these enzymes complement the previous action of pepsin and favor protein hydrolysis during the intestinal phase.^53^ In fact, β-lactoglobulin, which is present in the UFC evaluated in the present study, has been reported to contain 19 preferential cleavage sites in its native structure for proteolysis (17 lysine and 2 arginine residues), and 13 peptides have been identified as products of specific tryptic hydrolysis.^54^ Moreover, *in vitro* digestion of raclette-type cheese following the INFOGEST protocol has demonstrated an increased generation of small peptides (5–6 amino acids) at the end of the intestinal phase relative to the gastric phase, in agreement with the present findings.^55^ A more recent work focused on the *in vitro* digestion of Parmigiano Reggiano cheese, carried out by Cattivelli et al., mentioned that most of the identified peptides were released from β- and α_S1_-caseins.^56^ Additionally, besides the action of the intestinal proteolytic enzymes, the increase of small size peptides in the supernatants of gastrointestinal digests could also be influenced by the presence of bile salts. In this line, Díaz-Piñero et al. showed that bile salts are able to promote the diffusion of micellar casein proteins toward the digestion medium, hence promoting a greater degree of proteolysis.^57^

### 3.3 Antioxidant Activity of Cheese Digests

*In vitro* antioxidant assays are a valuable tool for a first approach to elucidating the bioactivity of food ingredients due to their sensitivity, speed, cost-effectiveness, and versatility. However, it is important to consider their limitations, such as the specificity of the free radicals used, errors caused by interfering compounds, or the lack of correlation with physiological redox processes, that are better represented in cell-based or *in vivo* models.^58^ The current recommendations indicate that both electron transfer (ET) and hydrogen atom transfer (HAT) assays should be used to draw a full picture of a biological sample.^59^ The ABTS (ET) assay and the ORAC (HAT) assay have been proven to be useful and suitable for the determination of the antioxidant capacity of different food matrices with diverse compositions, including milk.^60–62^ Therefore, these two methods were used to evaluate the potential antioxidant activity of the bioaccessible fraction (i.e., SN) of the UFC.

Figure 3A shows the TEAC values of SNs from gastric and gastrointestinal digests from all cheese types. Gastric SNs showed higher antioxidant activity compared to intestinal samples, as opposed to what was foreseen. Among the gastric samples, the greatest response was observed for W, followed by C, WM, and M, respectively. Even though W gastric SN showed more small size peptides (500–2000 Da) among gastric cheese digests, these impaired results could be produced by the interaction of the radical ABTS with other reducing compounds naturally present in fresh cheese gastric digests, such as sugars, free amino acids, or PL. Xie et al. emphasized the high antioxidant activity through the ABTS assay of *A. rolleston* starfish gonad lipids, which are composed of TG and PL rich in omega-3 FA, suggesting a potential ABTS radical scavenging activity of these types of lipids.^63^ Xu et al. mentioned that the FA composition of vegetable oil emulsions would exert great influence on their antioxidant activity.^64^ In this line, TG and PL that remain undigested during the gastric phase and are absent in the gastrointestinal digests could potentially interfere with the electron-donating mechanism underlying the ABTS assay.

**Figure 3:**
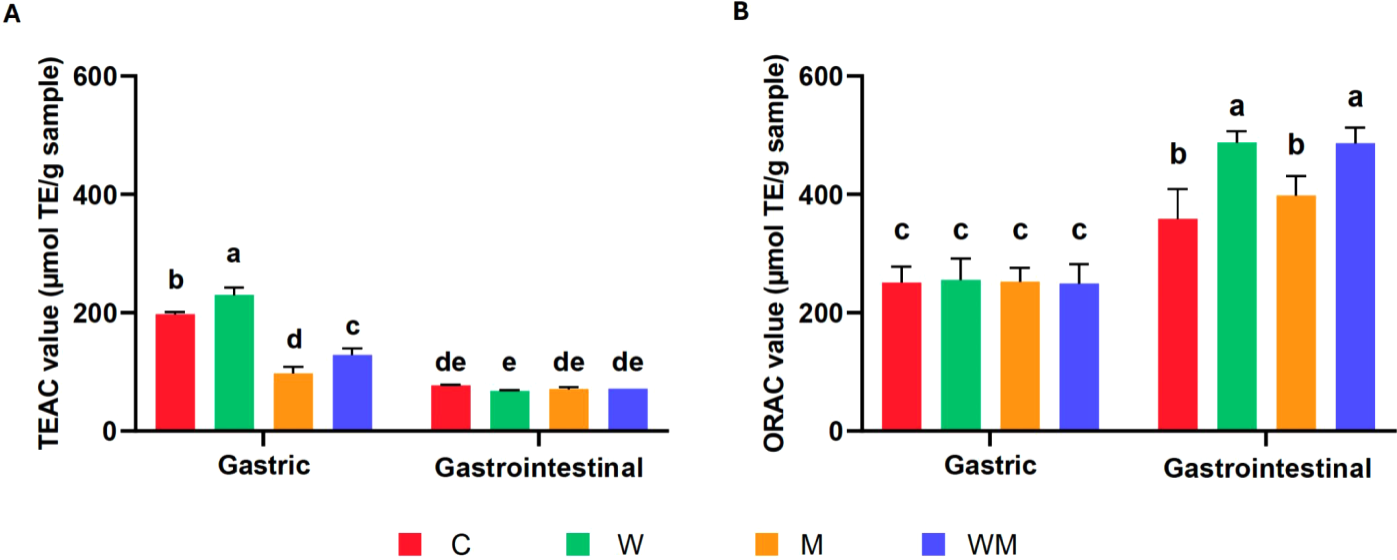
Antioxidant activity determination of supernatants from *in vitro* gastric and gastrointestinal digests of fresh cheeses made from ultrafiltered milk. A) ABTS assay. B) ORAC assay. C: control. W: ω-3 fatty acids (FAs). M: milk fat globule membrane (MFGM). WM: ω-3 FA and MFGM. Superscripts a, b, c, and d indicate significant differences between samples.

Figure 3B illustrates the ORAC values of SNs from gastric and gastrointestinal digests. Regarding gastric digests, no significant differences were observed among samples, all showing significantly lower values compared to gastrointestinal digests. These results might be related to the peptide profile, as gastrointestinal digests contain a higher relative abundance of small size peptides (<5 kDa, Figure 2 and Table 3), which have been previously associated with antioxidant activity.^7^ In this line, a simulated *in vitro* digestion study on buttermilk reported that peptides derived from β-casein, such as those with *m*/*z* of 604 and 915 Da (Table 3), would exert antioxidant activity.^65^ Calvante et al. identified, characterized, and evaluated both the antioxidant capacity and enterocyte transport of peptides derived from the tryptic hydrolysis of β-lactoglobulin.^52^ Notably, some of which were also detected in the present study with *m*/*z* of 543 and 656 Da (Table 3). A recent study by Ali et al. reported that the *in vitro* digestion of cheddar cheese also released intrinsic antioxidant peptides.^66^ In addition, other studies identified naturally occurring peptides with potential antioxidant capacity in Burgos-type cheese, derived from α_s1_- and β-casein, which could contribute to the increased ORAC values observed in the SN intestinal digests.^67,68^ Overall, these results indicate that UFC would possess inherent antioxidant activity due to the naturally present peptides derived from milk proteins.

Regarding antioxidant activity associated with cheese enrichments, the SNs obtained from W and WM gastrointestinal digests exhibited the highest ORAC values (488 ± 19 and 487 ± 26 μmol TE/g sample, respectively). This significant increase (*p* < 0.05) in antioxidant capacity should be attributed to the use of naturally enriched milk with ω-3 FA (Figure 3B). Few studies have used the ORAC assay to determine the antioxidant capacity of foods with different FA profiles, mainly focusing on vegetable oils.^69^ Interestingly, a previous study by Tijerina-Sáez et al., which used the ORAC assay on human milk samples, did not observe significant differences on the antioxidant capacity related to ω-3 FA.^70^ To the best of our knowledge, this is the first time that the increase in peroxyl radical scavenging capacity has been demonstrated in digests from ω-3 FA-enriched food products. However, the use of biochemical methods to assess the antioxidant activity in functional dairy products has been previously documented. For instance, a bioaccessibility study on a novel functional full-fat hard cheese demonstrated that the addition of nanoencapsulated catechins enhanced antioxidant capacity as measured by the ORAC assay.^71^ Moreover, the review by Stobiecka et al. compiles numerous studies employing various biochemical assays, including ABTS, for the characterization of enriched dairy products.^72^ Therefore, the methodology applied in this study aligns with previously established approaches. Nevertheless, transient changes during the gastro and/or intestinal phases, the effect of different digestive enzymes, and the digestive pH could possibly affect the bioaccessibility and bioavailability of the antioxidants present in digests.^73^ Thus, further *in vitro* digestion studies, combined with cellular transport studies and animal models, are encouraged to determine the bioavailability and bioactivity of the enriched UFC.

The present study represents an initial step toward understanding the digestion process and nutrient bioaccessibility of UFC, with a particular focus on the effects of ω-3 fatty acids and MFGM enrichment on protein and lipid behavior under simulated digestive conditions, as well as the release of antioxidant compounds. Neither protein nor lipid digestibility was negatively affected by either enrichment, as bioaccessible nutrients were detected after gastrointestinal digestion. Among the tested samples, WM digests exhibited the highest antioxidant capacity, which was associated with both the total number of peptides and the ω-3 FA content. Future work should identify the specific peptide sequences responsible for the observed antioxidant capacity, both before and after digestion, and evaluate other potential bioactivities. Combined with the prior comprehensive characterization of WM in Hueso et al.,^25^ our findings reinforce the potential of developing functional dairy products with favorable physicochemical, sensory, and digestion-related properties. If applied to other dairy products with comparable production, this approach could facilitate the design of products tailored to diverse consumer profiles and demands.

## Supplementary Material



## Notes

Tables S1 and S2 show the semiquantification (% band density) of identified protein bands after SDS-PAGE analysis for all UFC samples analyzed (PDF)

## Abbreviations

ABTS: 2,2′-azino-bis­(3-ethylbenzothiazoline-6-sulfonic) acid
C: control cheese
DG: diglyceride
ET: electron transfer
FA: fatty acid
FFA: free fatty acid
GluCer: glucosyl ceramide
HAT: hydrogen atom transfer
LacCer: lactosyl ceramide
M: MFGM concentrate enriched cheese
MFGM: milk fat globule membrane
MG: monoglyceride
ORAC: oxygen radical absorbance capacity
PC: phosphatidylcholine
PE: phosphatidylethanolamine
PL: polar lipid
PS: phosphatidylserine
SM: sphingomyelin
SN: supernatant
TEAC: Trolox equivalent antioxidant capacity
TG: triglyceride
UFC: fresh cheese made from ultrafiltered milk
W: ω-3 FA enriched cheese
WM: cheese enriched with both ω-3 FA and MFGM
