# Supplementary material for: In Vitro Gastrointestinal Digestion of Fresh Cheese: Effect of ω‑3 Fatty Acids and Milk Fat Globule Membrane Enrichment on Nutrient Digestibility and Antioxidant Activity
Source: J Agric Food Chem. 2025 Oct 25;73(44):28240–9. doi: 10.1021/acs.jafc.5c10823 (PMC12593394; doi:10.1021/acs.jafc.5c10823)
Supplement: Supplementary file 1 [file jf5c10823_si_001.pdf]

Supporting Information

In vitro gastrointestinal digestion of fresh cheese: effect of ω-3 fatty acids and milk fat globule membrane enrichment on nutrient digestibility and antioxidant activity

Diego Hueso[1], Samuel Paterson[1,2], Victoria Martínez-Sánchez[1,3], Blanca Hernández-Ledesma[1], Antonio Pérez-Gálvez[3], Javier Fontecha[1], Pilar Gómez-Cortés[1,*]

[1] Department of Bioactivity and Food Analysis, Institute of Food Science Research (CIAL, CSIC-UAM), Nicolás Cabrera 9, Madrid, 28049, Spain

[2] Department of Nutrition and Food Science, Faculty of Pharmacy, Complutense University of Madrid (UCM), Plaza Ramón y Cajal s/n, 28040 Madrid, Spain.

[3] Food Phytochemistry Department, Instituto de la Grasa (CSIC), Campus Universitario, Building 46, 41013, Sevilla, Spain.

Corresponding author: Pilar Gómez-Cortés (p.g.cortes@csic.es)

Supporting Table 1. Semi-quantification (% band density) of identified proteins bands after SDS-PAGE analysis of **C:** control cheese, **W:** ω-3 fatty acid enriched cheese, and their corresponding gastric (G) and gastrointestinal (GI) digests.

Milk proteins	App. MW (kDa)	**C**	**C** G SN[1]	**C** G P[2]	**C** GI SN	**C** GI P	**W**	**W** G SN	**W** G P	**W** GI SN	**W** GI P
Xanthine Oxidase / Dehydrogenase (XO/XDH)	155	2.3	-	-	-	-	1.7	-	-	-	-
Lactoferrine (LF)	80	4.8	-	-	-	-	1.7	-	-	-	-
Bovine Serum Albumin (BSA)	66.5	1.5	-	-	-	-	1	-	-	-	-
Butirophiline (BPH)	66	2.1	-	-	-	-	2.6	-	-	-	-
Adipophyline (ADPH)	48.1	10.4	-	-	-	-	9.3	-	-	-	-
Lactadherin (PAS-6/7)	47	5.2	-	-	-	-	5.4	-	-	-	-
α_{s2}-Casein (αs2-CN)	25.2	8.4	-	7.7	-	-	9.4	-	8.5	-	-
β-Casein (β-CN)	24	19.4	-	5.4	-	-	19.8	-	5.2	-	-
α_{s1}-Casein (αs2-CN)	23.6	19.3	-	1.1	-	-	26.4	-	2.4	-	-
κ-Casein (κ-CN)	19.0	2.9	-	2.4	-	-	1.6	-	3.2	-	-
β-Lactoglobulin A	18.4	5.2	11	-	-	-	4.6	11.9	-	-	-
β-Lactoglobulin B	18.2	4.3	47.6	-	-	-	4.5	2.38	-	-	-
para-κ-Casein (*para*-κ-CN)		9	-	20.9	-	-	9.5	-	25	-	-
α-Lactalbumin (α-LA)	14	2.3	29.8	-	-	-	1.4	46.1	-	-	-
Digestion enzimes											
Human salivary α-amylase	62	-	-	-	-	-	-	-	-	-	-
Pancreatic α-amylase	55.4	-	-	-	-	-	-	-	-	-	-
Pancreatic lipase (PL)	52	-	-	-	73	42.1	-	-	-	73.9	58.1
Pepsin	38.3	-	-	2.9	-	-	-	-	1.8	-	-
Chymotrypsin	27	-	-	-	15.2	4	-	-	-	17	3.3
Trypsin	24	-	-	-	6.7	-	-	-	-	4.2	-

[1]SN: Supernatant

[2]P: Pellet

Supporting Table 2. Semi-quantification (% band density) of identified proteins bands after SDS-PAGE analysis of **M**: cheese enriched in milk fat globule membrane (MFGM), **WM:** cheese enriched in both ω-3 fatty acids and MFGM, and their corresponding gastric (G) and gastrointestinal (GI) digests.

Milk proteins	App. MW (kDa)	**M**	**M** G SN[1]	**M** G P[2]	**M** GI SN	**M** GI P	**WM**	**WM** G SN	**WM** G P	**WM** GI SN	**WM** GI P
Xanthine Oxidase / Dehydrogenase (XO/XDH)	155	1.8	-	-	-	-	1.2	-	-	-	-
Lactoferrine (LF)	80	3.6	-	-	-	-	3	-	-	-	-
Bovine Serum Albumin (BSA)	66.5	1.2	-	-	-	-	1.1	-	-	-	-
Butirophiline (BPH)	66	1.8	-	-	-	-	2	-	-	-	-
Adipophyline (ADPH)	48.1	12.2	-	-	-	-	11.8	-	-	-	-
Lactadherin (PAS-6/7)	47	2.2	-	-	-	-	3.6	-	-	-	-
α_{s2}-Casein (αs2-CN)	25.2	9.1	-	4.3	-	-	10.2	-	0.2	-	-
β-Casein (β-CN)	24	18	-	11.5	-	-	18.7	-	4	-	-
α_{s1}-Casein (αs2-CN)	23.6	21.8	-	4.6	-	-	22.7	-	1.4	-	-
κ-Casein (κ-CN)	19.0	3.3	-	1.6	-	-	3	-	1.5	-	-
β-Lactoglobulin A	18.4	3.5	29.3	-	-	-	3.6	13.6	-	-	-
β-Lactoglobulin B	18.2	4.5	52	-	-	-	4.1	15.9	-	-	-
para-κ-Casein (*para*-κ-CN)		11.8	-	30.3	-	-	9.9	-	23.9	-	-
α-Lactalbumin (α-LA)	14.18	3.4	14.1	-	-	-	2.8	57.3	-	-	-
Digestion enzymes											
Human salivary α-amylase	62	-	-	-	-	-	-	-	-	-	-
Pancreatic α-amylase	55.4	-	-	-	-	-	-	-	-	-	-
Pancreatic lipase (PL)	52	-	-	-	59.2	45.3	-	-	-	61.2	49.6
Pepsin	38.3	-	-	2.1	10.1	0.8	-	-	1.4	5.9	8
Chymotrypsin	27	-	-	-	16.7	13.7	-	-	-	7.5	10.2
Trypsin	24	-	-	-	5.2	12.1	-	-	-	8.5	4.4

[1]SN: Supernatant

[2]P: Pellet
